# Notes and News

**Published:** 1890-03-22

**Authors:** 


### Notes and News.

Collected and Communicated.

The Medical Superintendents' Society held a meeting lately at the St. George's-in-the-East Infirmary, when the important question of the provision made in infirmaries for nurses during sickness was under consideration. Dr. Harris also showed some obscure cases of nervous disease. Altogether, the meeting was a most interesting one.

The Poor Children's Aid Committee, of Rochdale, issue their annual report of the Convalescent Home at St. Anne's-on-the-Sea. During the year 112 small convalescents had three weeks by the sea, and returned to Rochdale with fresh health and strength and a fund of pleasant memories. The home is economically managed, the total expenditure being £314.

Last Saturday afternoon the Princess of Wales, accompanied by the Princess Victoria and Prince George of Wales, and attended by Miss Knollys, visited the Royal Hospital for Children and Women, and distributed flowers to the patients. Last week Princess Beatrice visited the General Lying-In Hospital in York Road, and the Duke and Duchess of Fife visited the Middlesex Hospital. Lord Sandhurst did the honours of the Middlesex, and the Duke and Duchess were much pleased with all they saw, and very gracious to the patients. These Royal visits to hospitals do much good in calling attention to deserving charities, and in setting an example of sympathy with suffering, which ought to be widely followed.

Carlisle will hold Hospital Sunday on May 11th, and the money collected will be distributed on the basis of the relative expenditure of the institutions. The Cumberland Infirmary has just issued a good report. The number of in-patients admitted during the past year has been 660, which, with 63 remaining in the hospital at the end of the year 1888, makes a total of 723 cases. The number of out-patients treated during the past year has been 1,576. The average stay of in-patients has been 40·3 days, and the average daily occupation of beds 79·8. The average annual expense of the maintenance of each patient has been £24 10s. 9d., and for provisions only £17 13s. 8d., as against £25 5s. 3d. and £18 16s. 8d. respectively for the year 1888. The average total cost per patient per diem has been 2s. 5d. The ordinary income for the past year has amounted to £4,031 4s. 8d.

Philanthropists seem to have the needs of convalescents specially before them at present, and Mr. Cadbury, of Birmingham, has offered to the Birmingham and Midland Sanatorium Committee the sum of £30,000 for the purchase of Moseley Hall, his present residence, to furnish the buildings and lay out the land, about twenty acres in extent, for the purpose of a children's sanatorium. In the rules of management proposed it is provided that no stimulants shall be used except under medical orders; it is also suggested that ladies have a hand in the management of the home. Mr. Cadbury is evidently a practical philanthropist, and desires to make his house as "grateful and comforting" to the weak as his cocoa is already.

Pasteur is not infallible; he is undoubtedly a great man, but he has not yet brought his scheme for the prevention of hydrophobia to perfection. An inquest was held last week on Joseph Rankin, aged thirteen years, who was bitten by a mad dog on January 29th last, and was sent to Paris to be treated by M. Pasteur; he returned home, and did not exhibit any sign of illness until March 5th. He died on the following Monday. Dr. Cardwell stated that death resulted from hydrophobia, and a verdict to this effect was returned. A London correspondent hears that Mrs. Priestley is preparing an album for presentation to M. Pasteur, which is to contain the signatures of the most distinguished amongst his admirers in America and this country.

Among those present at the meeting of the Hospitals Association at Westminster Hospital on March 12 were: Dr. J. S. Bristowe, F.R.S., in the chair; Sir Edmund Hay Currie (reader of the paper), Dr. W. H. Blenkinsop, Dr. A. Brenchley (Camberwell Provident Dispensary), Mr. Charles H. Byers (Secretary, Metropolitan Hospital), Miss A. Glanfield Clark, Mr. Richard Davy, F.R.C.S. (Surgeon, Westminster Hospital), Rev. Dr. Finch, Mr. H. Nelson Hardy, F.R.C.S., Mr. E. Murray Ind (Chairman of the London Hospital), Rev. James Mahomed (Chaplain, London Hospital), Mr. P. Michelli (Secretary, Seamen's Hospital, Greenwich), Mr. S. M. Quennell (Secretary, Westminster Hospital), Dr. T. Gilbart Smith, Dr. O. Sturges (Physician, Westminster Hospital), Mr. John Tweedy, F.R.C.S. (Ophthalmic Surgeon, University College Hospital), and Miss Vincent.

It is a debatable point whether certain societies for the suppression of cruelty do not cause more suffering than they suppress. Have we not all seen a drawing-room full of highly sensitive women weeping over a vivid picture of the wrongs of some poor donkey? Now the donkey is not a nervous animal and does not long remember its injuries, but many of those women probably did not sleep well for nights, so great was their sympathy with suffering. Again, the painful publications of these societies, full of horrible stories, are circulated widely, and throw a gloom over many a young girl's mind. Shilling shockers are sometimes entirely effaced when compared with the blood and murder tales of magazines with such innocent titles as the *Child's Guardian*. Even in the cause of charity and of the suppression of cruelty it is wrong to dwell unduly on the wickednesses of this world, and to try and tinge the imaginative minds of highly-cultured women with the pessimism bred of a belief in universal suffering. All children and all animals are not ill-treated, and those that are ill-treated should be protected by men, not moaned over by women. Of course, there are a certain class of women who can work well in this field, but it is not to them that these highly-coloured tales are told—it is to the enervated dweller in drawing-rooms whose purse is heavy, and whose mind is weak. It is so much easier to entice a guinea from such a one! What would Mr. Waugh say if an action were brought against him for cruelty to women?

The following vacancies are announced this week, the amount of the salary in each case being given after the name of the institution:

**Resident Medical Officers.**

Radcliffe Infirmary, House Physician—£80, board, etc.
Hospital for Diseases of the Throat, Golden Square, Resident Medical Officer—£50, board, etc.
St. Peter's Hospital, House Surgeon—£25, board, etc.
Birmingham General Hospital, Assistant House Surgeon—Board, etc.
Government Analyst and Professor of Chemistry for Trinidad—£600.
South Devon Hospital, Assistant House Surgeon—Board, etc.
Wrexham Infirmary, House Surgeon—£80, board, etc.
Liverpool Infirmary for Children, Assistant House Surgeon—Board, etc.
Rangoon Municipality, Health Officer—Rs. 600 per mensem.

**Non-Resident Medical Officers.**

Kensington Dispensary, Honorary Medical Officer.
West London Hospital, Surgeon-Dentist.
Royal Hospital for Children and Women—Registrar and Chloroformist—£20.

Mr. H. W. Crosse has issued a little sixpenny handbook, called "Sanitary Aids to House-Hunters," which is worthy of notice because of the numerous illustrations. Amongst the poorer class of house-hunters the slightest sketch is more easy of comprehension than pages of type, and the illustrations to this small book are remarkably clear and need but little explanation. The book is published by Upcott Gill.

Another munificent gift to a medical charity. Lady Howard de Walden, who is now occupying The Mote, Earl Romney's seat near Maidstone, has expressed her intention of endowing, at a cost of £10,000 or £12,000, a ward at the West Kent Hospital at Maidstone, as a thank-offering for her recent recovery from a serious illness. Her ladyship has also intimated her desire to endow a curacy for St. Philip's Church, Maidstone.

The Home for Sick Children at Southsea is neither a hospital nor an ordinary convalescent institution; it grants to such of its inmates as require it medical treatment, and does not insist that a patient must be up and about. The expenditure last year was £618, and £200 of consols had to be sold. The patients numbered 87. Comparing accounts is rather hopeless work, but we cannot help noticing that the expenditure at Southsea for 87 patients is almost double that at St. Anne's-on-Sea for 112 patients, save that at Southsea a balance of £124 is carried over.

At the annual meeting of the Charity Organization Society, on March 12th, a resolution was adopted urging the Govern- to appoint a Royal Commission or Select Committee of the House of Lords to make inquiry in regard to the financial and general management and common organization of medical institutions, endowed and voluntary, and in regard to the administration of poor-law institutions for the aid of the sick in the metropolis, and to make recommendations thereon. Mr. R. Brudenell-Carter, F.R.C.S., moved the resolution, and said that £100,000 was spent annually in appeals. The Rev. H. L. Paget seconded the motion, which was carried.

Dr. Forbes Winslow has added to existing institutions by opening an out-patient department in Euston Square, where he proposes to prescribe for mental cases amongst the poor. In the course of his speech Dr. Winslow said, "Large numbers of murders and suicides were committed in London by persons while suffering from various forms of dangerous lunacy, which might by treatment in its earlier stages have been cured, and it was hoped that in that direction the new hospital, which at present would be confined to the treatment of out-patients, would do much good." This looks as though the new hospital were nothing more nor less than a trap to catch Jack the Ripper.

A correspondent writes: I have noticed your annotation about "The Lady and the Doctor" upon page 360 of last week's Hospital, and write to state that the legislation desired is now in operation. There is a special section in the Lunacy Amendment Act, which was passed last session, to relieve a medical man from responsibility in connexion with lunacy certificates, "if such person has acted in good faith and with reasonable care." It is Section 12, and though the Act does not come into operation until May 1st, immediate relief was to be given to medical men. "This section shall come into force immediately after the passing of this Act."

The report of the Hospital of SS. John and Elizabeth, 50, Great Ormond Street, W.C., is satisfactory, and should commend this charity to the liberal support of the benevolent. The special work of this hospital is to benefit particular classes of patients for whom the usual hospitals are not available. To this class belong the incurable cases and patients suffering from diseases not usually incurable, but which require for their complete relief a long continued rest and treatment. 145 such cases were treated during 1889, of which 70 were cured, 25 died, and 50 remained under treatment. A benevolent person with means in search of a good object should visit this hospital. £25 a year entitles a subscriber to one paid patient in the hospital always, and an endowment of £500 gives the privilege of one bed in perpetuity. There are no out-patients, a notable fact worthy of all praise.

Workhouse infirmaries are a sore subject to such of us as are used to the method of hospital life, for in many of these infirmaries, especially the smaller ones, the care of the sick seems of minor importance. Several cuttings from the *Halifax Guardian* have reached us, which show that at the Halifax Workhouse the house committee dine weekly with the master and matron, which must cause a laxity of discipline. Also this enlightened committee have decided, contrary to the advice of the doctor, that sick paupers need no care from paid nurses at night. At Richmond Workhouse a night nurse was lately introduced, but all sorts of charges were brought against her by those who preferred the old order of things, and though these charges were not proven before the committee, it is probable that her position will be made so unpleasant that she will be obliged to leave. The Workhouse Infirmary Nursing Association has done a great and good work, and we heartily wish that all infirmaries were supplied with the excellent nurses it trains, then there would be fewer stories like the above.

The new journalism is slowly but surely penetrating into medical as well as other literature, and, with all its faults and all its virtues, it is bringing hospital questions before the great public. Here are some specimens of what local papers can do, in their efforts to rival the startling headings of American journals:—

DR. DAVIES-COLLEY'S SUCCESSOR
AT CHESTER INFIRMARY.

RESULT OF THE BALLOT: THE TORIES
CARRY THEIR MAN.

This is from Chester; the following is from Aberdeen:—

**Among the Sick Children.**

Year's Work at the Hospital.

295 CHILDREN CURED.

A few years ago we should have regarded this as undignified, but now we sigh and submit, and find our consolation in the increased interest shown in our charities by the readers of the new journalism.

